# Protective Role of Omega-3 Polyunsaturated Fatty Acid against Lead Acetate-Induced Toxicity in Liver and Kidney of Female Rats

**DOI:** 10.1155/2014/435857

**Published:** 2014-06-18

**Authors:** Heba M. Abdou, Mohamed A. Hassan

**Affiliations:** ^1^Zoology Department, Faculty of Science, Alexandria University, Moharram Bey, Alexandria 21511, Egypt; ^2^Protein Research Department, Genetic Engineering and Biotechnology Research Institute (GEBRI), City of Scientific Research and Technological Applications (SRTA-City), New Borg El-Arab, Alexandria 21934, Egypt

## Abstract

The present study was conducted to investigate the protective role of Omega-3 polyunsaturated fatty acids against lead acetate-induced toxicity in liver and kidney of female rats. Animals were divided into four equal groups; group 1 served as control while groups 2 and 3 were treated orally with Omega-3 fatty acids at doses of 125 and 260 mg/kg body weight, respectively, for 10 days. These groups were also injected with lead acetate (25 mg/kg body weight) during the last 5 days. Group 4 was treated only with lead acetate for 5 days and served as positive control group. Lead acetate increased oxidative stress through an elevation in MDA associated with depletion in antioxidant enzymes activities in the tissues. Moreover, the elevation of serum enzymes activities (ALT, AST, ALP, and LDH) and the levels of urea and creatinine were estimated but total proteins were decreased. Also, lead acetate-treatment induced hyperlipidemia via increasing of lipid profiles associated with decline in HDL-c level. Significant changes of Hb, PCV, RBCs, PLT, and WBCs in group 4 were recorded. The biochemical alterations of lead acetate were confirmed by histopathological changes and DNA damage. The administration of Omega-3 provided significant protection against lead acetate toxicity.

## 1. Introduction

Lead is one of mankind's oldest environmental and occupational toxins [1]. The exposure to lead can occur from a multitude of sources such as soil, air, water, and industrial pollutants. There are, worldwide, six categories of products considered as source of lead exposure, that is, gasoline additives, food can soldering, lead based paints, ceramic glazes, drinking water systems, and folk remedies [2]. Health hazards from increased lead exposure as a result of industrial and environmental pollution are recognized. It has been found to cause a wide range of biochemical and physiological dysfunctions [3]. Moreover, the long term lead exposure generates reactive oxygen species and different free radicals. Also, it inhibits antioxidant enzymes activities, such as superoxide dismutase (SOD) and catalase (CAT), while it decreases the level of glutathione [4, 5]. Lead induced oxidative damage in the kidney as evidenced by enhancement of lipid peroxidation [6, 7]. Lead is a highly poisonous environmental pollutant and is known to affect organs like liver, kidney, blood, and central nervous system of mammals [8, 9]. Several reports have indicated that lead can cause neurological, hematological, gastrointestinal, reproductive, circulatory, and immunological pathologies [10, 11]. Omega-3 fatty acids (Omega-3 FAs) are considered as strong antioxidants and their role as anticancer agent has been extensively confirmed in most of the human malignancies [12, 13]. Furthermore, the anti-inflammatory potential of long chain Omega-3 FAs in many chronic diseases has been suggested [14, 15]. The role of Omega-3 FAs in inhibiting proliferation, inducing apoptosis, and promoting differentiation in many cancers has been recently studied [16, 17]. In addition, another finding indicates that Omega-3 FAs act synergistically with certain chemotherapeutic agents [18]. Omega-3 FAs were found to play protective roles in the liver, cardiovascular system, and kidney and they have been widely used in clinical peroperative total parenteral nutrition [19, 20]. Therefore, the present study was carried out to investigate the protective effects of Omega-3 FAs against lead acetate-induced oxidative stress, biochemical changes, and DNA damage.

## 2. Materials and Methods

### 2.1. Chemicals

Lead acetate was purchased from Merck (Germany).

Omega-3 was purchased from Efamol Ltd., 14 The Mole Business Park Leatherhead, Surrey, KT227BA, UK in the South East of England. All other chemical materials that were used in this study were purchased from Sigma Chemical Co., (St. Louis, MO, USA).

### 2.2. Animals and Experimental Design

Twenty-eight adult Wistar albino female rats (weighting 170–200 gm) were obtained from the animal house of Faculty of Medicine, Alexandria University, Egypt. The local committee approved the design of the experiments and the protocols were carried out according to the guidelines of the National Institutes of Health (NIH). Rats were housed in stainless steel cages placed in a well-ventilated rat house, maintained for two weeks as acclimatization period under standard laboratory conditions on free supply of food and water provided* ad libitum,* and subjected to natural light for 12 hrs and dark for 12 hrs cycles. After the period of acclimatization, rats were divided randomly into four groups, 7 animals in each. The animal experiments were conducted for 10 days. Group 1 was injected daily with 0.5 mL of saline solution (0.9% NaCl) i.p. for 10 days and was used as negative control (−ve). Groups 2 and 3 were administrated orally with two doses of Omega-3 (125 and 260 mg/kg body weight, resp.) by gavage for the first five days as protective agent [21]. These groups were injected (i.p.) by 0.5 mL of lead acetate at a dose of 25 mg/kg body weight/day for the other 5 days in combination with Omega-3. Group 4 was injected (i.p.) by 0.5 mL lead acetate only at a dose of 25 mg/kg body weight/day for the last 5 days of the experiment and was used as positive control (+ve) according to Ponce-Canchihuamán et al. [22].

### 2.3. Blood Collection and Tissue Preparation

At the end of treatment, rats fasted for 12 hrs before being anesthetized and sacrificed by cervical dislocation. Blood samples were collected from the sacrificed animals and left in refrigerator for 30 min before centrifugation. The clear nonhemolyzed sera were stored at −20°C till measurements. However, heparin was used as an anticoagulant and noncoagulated blood was tested shortly after collection for applying in determination of hemoglobin (Hb), packed cells volume (PCV), red blood cells (RBCs) count, white blood cells (WBCs), and platelets (PLT) count by particle counter (ERMA Inc., Tokyo, model PCE-210).

Liver and kidney were immediately removed and washed using chilled saline solution. These tissues were minced and separately homogenized (10% w/v) using a homogenizer (Potter-Elvehjem) in ice-cold sodium potassium phosphate buffer (0.01 M, pH 7.4) containing 1.15% of KCl. The homogenates were centrifuged at 10,000 ×g for 20 min at 4°C and the supernatant was used for assaying of the enzymes activities.

### 2.4. Biochemical Analysis

Stored serum samples were analyzed for the activities of aspartate aminotransferase (AST; EC 2.6.1.1), alanine aminotransferase (ALT; EC 2.6.1.2), alkaline phosphatase (AlP; EC 3.1.3.1), and lactate dehydrogenase (LDH; EC 1.1.1.27) which were determined using kits from Sentinel Ch. (via principle Eugenio 5-20155 Milan, Italy). Also, serum total protein, albumin, urea, creatinine, cholesterol, total lipids, triglycerides, HDLC, and low density lipoprotein (LDLC) were determined using kits from Sentinel Ch. (via principle Eugenio 5-20155 Milan, Italy). The lipid peroxidation end product, MDA, was measured as thiobarbituric acid reactive substance. Also, the levels of GSH and the activities of antioxidant enzymes including the catalase enzyme (CAT; EC 1.11.1.6), superoxide dismutase (SOD, EC.1.15.1.1), and glutathione peroxidase (GPx; EC. 1.1.1.9) were assayed using commercial assay kits according to the manufacturer's instructions.

### 2.5. Histopathology

Specimens of liver tissues were immediately fixed in 10% formalin, treated with conventional grade of alcohol and xylol, embedded in paraffin, and sectioned at 4–6 *μ* thickness. The sections were stained with Haematoxylin and Eosin (H&E) stain for studying the histopathological changes [23].

### 2.6. Random Amplified Polymorphic DNA Technique (RAPD)

#### 2.6.1. Extraction of DNA

DNA was extracted from livers of the four groups following the method described by Bardakci and Skibinski [24].

#### 2.6.2. Polymerase Chain Reaction (PCR) Primers

In the present work, ten-base long oligonucleotides primers were used to initiate the PCR amplifications. Primers were randomly selected on the basis of GC content and annealing temperature for RAPD-PCR amplification as in Table 1.

#### 2.6.3. PCR Amplification and Agarose Gel Electrophoresis

PCR amplifications were performed according to the procedure described by Williams et al. [25] using the isolated DNA from three hepatic samples of each group.

#### 2.6.4. Agarose Gel Electrophoresis

The amplified DNA fragments were separated on 1.5% agarose gel and stained with ethidium bromide. DNA ladder in range (100 bp–3000 bp) was used in this study as marker for amplified pattern. The amplified pattern was visualized and photographed by gel documentation system.

### 2.7. Statistical Analysis

The data entry was done into a binary data matrix as discrete variables and was analyzed according to Steel and Torrie [26]. Statistical significance of the difference in values of control and treated animals was calculated by (*F*) test at 5% significance level. Data of the present study were statistically analyzed by using Duncan's multiple range test (SAS, 1986). All RAPD profiles were analyzed using stat program which showed the similarity between the amplified PCR products. The best amplified PCR product of each group was selected to compare between them at genetic levels.

## 3. Results

### 3.1. Biochemical Parameters

The results showed that the treatment with lead acetate significantly (*P* < 0.05) increased serum AST, ALT, AlP, and LDH compared to the control (Table 2). On the other hand, data indicated that the serum total proteins and albumin were significantly (*P* < 0.05) decreased after lead acetate treatment compared to the control group. Meanwhile, serum AST, ALT, AlP, total proteins, and albumin were normalized after treatment with either of the two doses of Omega-3 (125 mg/kg or 260 mg/kg body weight) in combination with lead acetate, compared to the lead acetate treated group (Table 2). Also, Table 2 indicated that the levels of serum urea, creatinine, and MDA were significantly (*P* < 0.05) increased in the lead acetate treated rats compared to the control ones reflecting renal impairment. On the other hand, treatment with lead acetate significantly (*P* < 0.05) decreased the activities of GPx, CAT, and SOD and the level of reduced GSH while it increased MDA level in both liver and kidney extracts compared to the control group (Table 3). Pretreatment of rats with Omega-3 (125 or 260 mg/kg body weight) prior to and during the injection with lead acetate ameliorated these parameters to reach the normal level. Furthermore, the dose of 260 mg/kg body weight was more effective than the dose of 125 mg/kg body weight in increasing the activities of SOD and GPx in the extracts of the liver and kidney.

The present data indicated that the serum total lipids, cholesterol, triglycerides, and LDL-c were significantly (*P* < 0.05) increased by lead acetate treatment, while HDL-c levels were decreased (Table 4). The other striking finding in the present study is that Omega-3 at both doses (125 or 260 mg/kg body weight) nearly normalized the lipid profiles in the serum of rats and became similar to the control values (Table 4).

### 3.2. Hematological Analysis

Hematological parameters revealed that the Hb and PCV values and the RBCs and PLT counts were significantly decreased (*P* < 0.05) in lead acetate treated group compared to the negative control group (Table 5). However, the results exhibited that Omega-3 at both doses (125 or 260 mg/kg body weight) nearly normalized the hematological parameters to become similar to the normal values (Table 5). On the other hand, WBCs count in lead acetate treated rats were significantly (*P* < 0.05) elevated as compared with the control group. However, treatment with Omega-3 at a dose of 260 mg/kg body weight was more effective than the other dose in normalizing the WBCs count.

### 3.3. Histopathological Investigations

**Figure 1:**
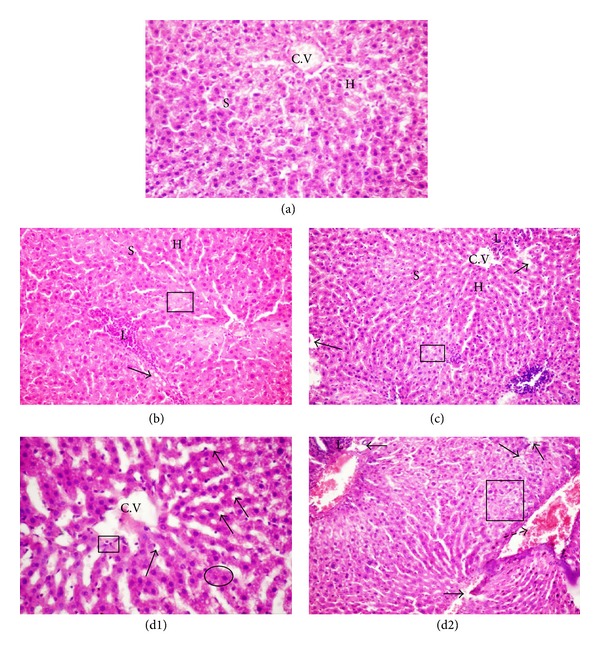
Paraffin sections stained by haematoxylin and eosin (H&E, ×200) for histopathological examination of liver tissues of rats as follows: control group (a) showing normal hepatocytes architecture (H), central vein (C.V), and normal blood sinusoids (S); (b) liver tissue of group 2 (lead acetate + Omega-3, 125 mg/kg body weight); (c) liver tissue of group 3 (lead acetate + Omega-3, 260 mg/kg body weight) showing histological alterations induced by lead acetate that were markedly reduced in groups 2 and 3. Liver tissue of group 4 (lead acetate treated rats) (d1 and d2) showing distended and hemorrhage in the portal vein (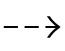), loss of the normal architecture, degenerated hepatocytes with pyknotic nuclei (□), and degenerated hepatocytes with vacuolated cytoplasm (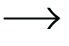). Condensed nuclei (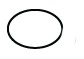) and lymphocytes aggregation (L) inside the hepatic tissue.

**Figure 2:**
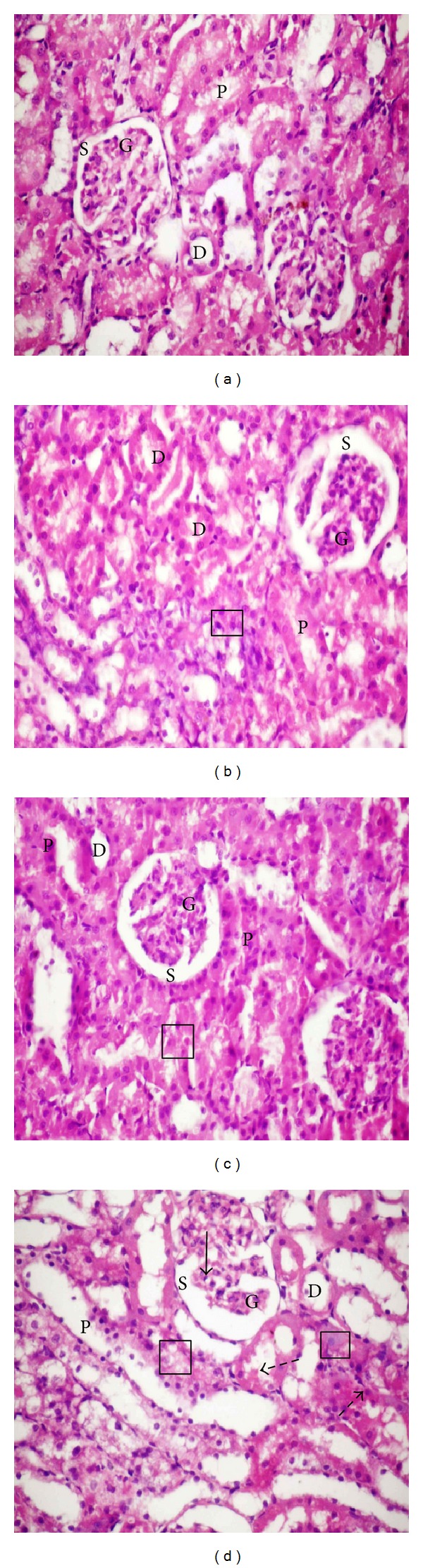
Paraffin sections stained by haematoxylin and eosin (H&E, ×200) for histopathological examination of the kidney tissue of rats treated as follows: control group (a); lead acetate plus Omega-3, 125 mg/kg body weight group (b); lead acetate plus Omega-3, 260 mg/kg body weight group (c). Kidney tissue of lead acetate treated rats (d) showing disruption of Bowman's capsule, shrunken glomeruli G with the capsular space S, and cytoplasmic pyknosis of some nuclei (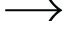), The degenerative changes in the epithelial cells lining the renal tubules (□), hemorrhagic area (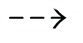) in renal tubules, and dilation in the renal tubules are compared to normal histological structure of the glomerulus and tubules in control group (a). Histological alterations induced in lead acetate at both doses of Omega-3 groups (b) and (c) were markedly reduced.

The histopathological studies of rats' livers are represented in (Figure 1). The light micrographs of liver tissues demonstrated normal hepatocytes in the control group showed normal hepatic architecture with distinct hepatic cells, sinusoidal spaces, and a central vein (Figure 1(a)), while livers of rats treated with lead acetate (group 4) showed loss of cellular architecture with dilatation of blood sinusoids, hemorrhage in the portal vein, degenerated hepatocytes with pyknotic nuclei, and vacuolated cytoplasm lymphocytes aggregation inside the hepatic tissue as in Figures 1(d1) and 1(d2). However, livers of rats treated with lead acetate plus Omega-3, 125 mg/kg (group 2), and rats treated with lead acetate plus Omega-3, 260 mg/kg (group 3), revealed that most of the histological alterations induced in lead acetate treated groups were markedly reduced (Figures 1(b) and 1(c)). Meanwhile, lead acetate treatment induced severe histopathological changes in the kidney tissues (Figure 2(d)) such as swelling of convoluted tubules, disruption of Bowman's capsule, shrunken glomeruli with the capsular space, cytoplasmic pyknosis of some nuclei, destruction of the epithelium lining the tubules, hemorrhagic area in renal tubules, and dilation in the renal tubules compared to normal histological structure. On the other hand, the histopathological studies of the kidneys of the control rats revealed normal glomerulus surrounded by the Bowman's capsule and proximal and distal convoluted tubules without any inflammatory changes (Figure 2(a)). Treatment with Omega-3 at both doses before and in combination with lead acetate slightly improved the kidney histology but extravasation of blood element with dilation of some proximal and distal tubules was still present as well as, presence of some glomeruli with the capsular space (Figures 2(b) and 2(c)).

### 3.4. Genetic Analysis Using RAPD-PCR

**Figure 3:**
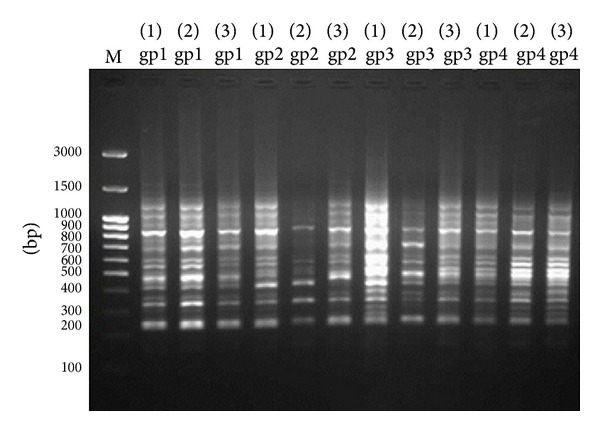
Pattern RAPD-PCR (primer 1) of hepatic DNA samples exposed to lead acetate and treated with two doses of Omega-3 using primer 1. The DNA ladder is in lane (M); lanes (gp1) represent group 1 (−ve control), lanes (gp2) group 2 (Omega-3 with dose 125 mg/kg body weight and lead acetate), lanes (gp3) group 3 (Omega-3 with dose 260 mg/kg body weight and lead acetate), and lanes (gp4) group 4 (+ve group).

**Figure 4:**
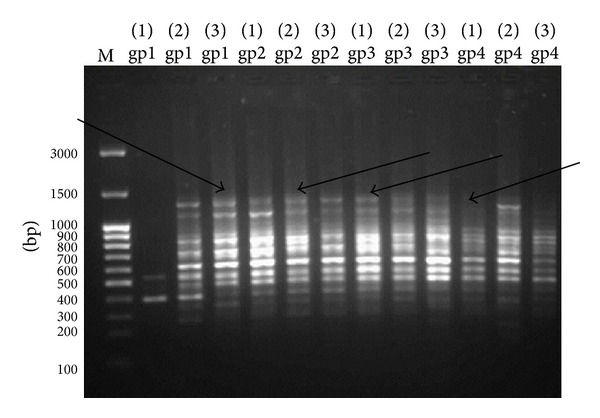
Pattern RAPD-PCR (primer 2) of hepatic DNA samples exposed to lead acetate and treated with two doses of Omega-3 using primer 2. The DNA ladder is in lane (M); lanes (gp1) represent group 1 (−ve control), lanes (gp2) group 2 (Omega-3 with dose 125 mg/kg body weight and lead acetate), lanes (gp3) group 3 (Omega-3 with dose 260 mg/kg body weight and lead acetate), and lanes (gp4) group 4 (+ve group). Arrows indicate loss of some amplification products of different groups.

**Figure 5:**
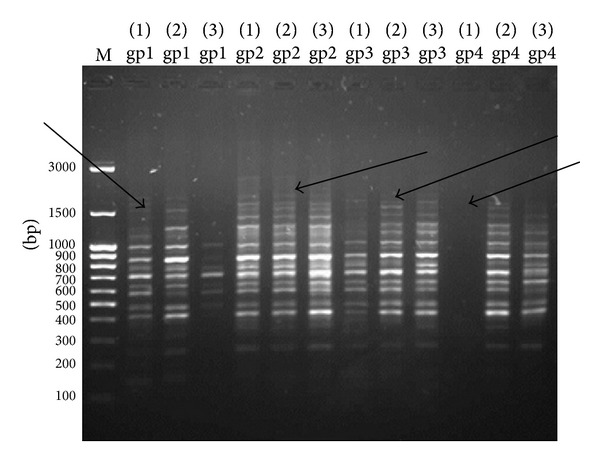
Pattern RAPD-PCR (primer 3) of hepatic DNA samples exposed to lead acetate and treated with two doses of Omega-3 using primer 3. The DNA ladder is in lane (M); lanes (gp1) represent group 1 (−ve control), lanes (gp2) group 2 (Omega-3 with dose 125 mg/kg body weight and lead acetate), lanes (gp3) group 3 (Omega-3 with dose 260 mg/kg body weight and lead acetate), and lanes (gp4) group 4 (+ve group). Arrows indicate loss of some amplification products of different groups.

Three of 10-mer. primers were used for investigating the significant changes of the DNA isolated from liver tissues. The three primers produced clear, sharp, monomorphic, and polymorphic bands as in Figures 3, 4, and 5. Primer 1 gave band patterns of almost the same profile between the three amplified samples of each group so it did not clarify any difference (Figure 3). In contrast, the other primers (primer 2 and primer 3) were most informative and they produced reproducible and the most distinguishable banding profiles between the amplified samples of each group after RAPD assays as in Figures 4 and 5. The amplified fragments of PCR products were summarized as in Table 6. Primers 2 and 3 produced highly similar RAPD fingerprints for negative control group (group 1) and groups 2 and 3 while they detected some changes in hepatic DNA of lead acetate treated group (group 4). We observed similar RAPD-PCR fingerprint using primer 2 in all 12 samples from the different groups as in Figure 3. In Figure 4, the amplified RAPD products of group 4 using primer 2 lost some bands of their three samples compared to the samples of negative control group and treated groups with Omega-3. Moreover the pattern showed a smear in the beginning of lanes 1 and 3 of group 4. The PCR products using primer 2 gave obvious results through the bands profile which were begun to appear at about 900 bp while the patterns of other groups were started at about 1350 bp. In addition, the RAPD-PCR using primer 3 did not amplify the first sample of group 4 (lane 1 of group 4) while lanes 2 and 3 of group 4 lost their bands at approximately 1650 bp compared to other groups as shown in Figure 5. The amplification products obtained by this method showed the presence of numerous bands from 250 to 1300 bp with primer 1, 300 to 1500 bp with primer 2, and 250 to 1700 bp with primer 3, respectively (Figures 3, 4, and 5).

**Figure 6:**
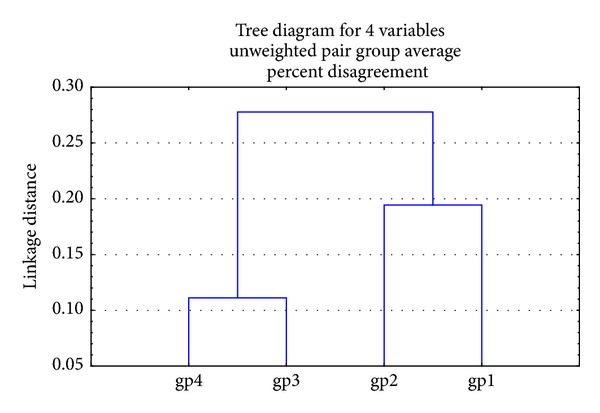
Dendrogram of the four applied groups generated by UPGMA based on 3 RAPD primers, where gp1 is group 1, gp2 is group 2, gp3 is group 3, and gp4 is group 4.

The RAPD products were scored as present (1) or absent (0) for each primer-genotype combination. The results of RAPD patterns of the 3 primers were summarized as in Table 6. Thirty-six bands were scored where 33 were polymorphic and 3 of them were monomorphic. Jaccard's coefficient of similarity was measured and a dendrogram (Figure 6) based on similarity coefficients was generated by using unweighted pair group method with arithmetic mean (UPGMA). The best amplified PCR was selected from each group to compare between them using stat software. The analysis of the results described the similarity between different samples of liver tissues (Table 7). The similarity of positive control (group 4) and treated group with Omega-3 (group 2) was about 64% and 81%, respectively, compared to negative control (group 1). The variations of the RAPD profiles of treated Omega-3 groups were compared to the negative and positive control groups.

## 4. Discussion

Lead has been known to be an environmental pollutant and its toxicity has also been associated with health hazards [8]. The liver acts as chief player in detoxification process and is one of the target organs affected by lead toxicity owing to its storage in the liver. Data shown in Table 2 demonstrated that treatment with lead acetate caused a significant elevation in the activities of liver enzymes AST, ALT, ALP, and LDH in serum confirming the histological damage shown in the liver (Figure 1). The present results revealed a significant increase in ALT, AST, and ALP in serum of lead acetate treated rats compared with negative control group. These results are in agreement with the results of Herman et al. [27], Ibrahim et al. [28], and Mehana et al. [29]. However, the activities of LDH were significantly elevated in serum of lead acetate treated rats and this result is similar to that of Ibrahim et al. [28]. The increasing of LDH in serum of lead acetate treated group may be due to spill out of this enzyme from the liver cytosol into the blood stream and/or liver dysfunction and disturbance in the biosynthesis of this enzyme with alteration in the permeability of liver membrane according to Yousef [30]. Also, Gaskill et al. [31] reported that releasing of AST, ALT, and LDH from the cell cytosol can occur as secondary changes to cellular necrosis. In addition, significant decrease in the total proteins and albumin in the serum of lead acetate treated group was compared to control group. Liver synthesizes proteins, among which is albumin, and the decrease in the total proteins and albumin levels in liver could be attributed to changes in protein and free amino acids metabolism and their synthesis in the liver [31]. This adverse effect might be caused by the interference of lead with protein synthesis or by the binding of lead to some metal-binding proteins and their removal through detoxification processes [30]. In an attempt to clarify the mechanism involved, it has been reported that lead caused a disruption in protein and RNA synthesis. Also, the observed decrease in the total proteins and albumin in the liver could be attributed to the damaging effect of lead acetate on liver cells as confirmed by increasing in the activities of serum AST and ALT (Table 2) after treatment of rats with lead acetate [32]. In the current study, the induced elevation of albumin, urea, and creatinine due to lead acetate administration indicated that the kidney function was affected. In addition, lead acetate caused a significant elevation in serum urea and creatinine reflecting renal impairment that is coinciding with histological damage of the kidney as shown in Figure 2 [33, 34]. It can be concluded that oxidative damages may be the primary cause of lead toxicity leading to lipid peroxidation and cellular damage. Thus, the obvious change in liver and kidney functions is related to the intensity of cellular damage. It has been shown that lead acetate undergoes metabolism in liver via esoteric and oxidative pathways generating elevated MDA levels that lead to hepatic necrosis [31]. The increased levels of MDA in the present study are associated with a reduced level of GSH and increased activities of serum enzymes which indicated the occurrence of an oxidative insult that caused hepatic and renal damage. Moreover, the toxicity with lead acetate in rats of group 4 leads to depletion of GPx, CAT, and SOD enzymes activities in liver and kidney (Tables 2 and 3) and these results are matching with the results which were achieved in a previous study [10]. The possible explanation could be related to the proposed role of GSH in the active excretion of lead through bile by binding to the thiol group of GSH and then being excreted. A decrease in GSH levels could lead to oxidative stress and a consequent increase in lipid peroxidation [7]. The presence of lipid peroxidation was observed in the current study due to decrease of SOD and CAT activities [10]. Enzymes, such as GPx, CAT, and SOD may contribute to the explanation of the mechanisms responsible for the decrease in GSH concentration in liver and kidney due to the exposure to this heavy metal [22].

The chemoprotective effect of Omega-3 on liver tissue was confirmed by the attenuation of the activities of serum ALT, AST, ALP, and LDH in addition to the normalization of serum protein and albumin contents (Table 2). These results are consistent with the results of Attaia et al. [35]. The mode of action of Omega-3 can be intercepted pharmacologically at different levels with agents that scavenge free reactive oxygen, block their generation, or enhance endogenous antioxidant capabilities [35].

The current results also indicated that treatment with Omega-3 decreased the level of MDA associated with an elevation in SOD and CAT activities, as well as in GSH content, in groups 2 and 3. The decrease in the MDA level by Omega-3 may be due to its antioxidant properties that inhibited lipid peroxidation [36] and this action helps stabilize the reactive radicals, preserve the cellular integrity, and restrain the severity of lead acetate. GSH plays a key role in many cellular processes involving protection of cells against oxidative stress, xenobiotics, and radiation and it is abundant with low molecular weight intracellular thiol [37]. In our study, Omega-3 prevented the decrement of GSH level suggesting that Omega-3 may protect the SH group of GSH from the reactive radicals that are produced from lead acetate toxicity. Similarly, Attaia and Nasr [38] found that Omega-3 could maintain normal levels of SOD and CAT activities. The antioxidant and anti-inflammatory effects of Omega-3 through scavenging of free radicals and inhibiting lipid peroxidation have been reported previously by Pauwels and Kostkiewicz [36]. This oxidant/antioxidant theory may explain the protective role of Omega-3 fatty acids against the hepatotoxicity and nephrotoxicity of lead acetate.

In the present study, a significant increase in serum total lipids, cholesterol, triglycerides, and LDL-c and a significant decrease of HDL-c of the rats treated with lead acetate were estimated (Table 4). HDL-c helps to scavenge cholesterol from extrahepatic tissues and the decrease of HDL-c concentration as in this study contributed to increasing cholesterol levels. There is evidence linking increased serum cholesterol and LDL-c levels to a higher risk for developing coronary heart diseases [39]. The present results exhibited that there was a significant decrease in serum total lipids, cholesterol, triglycerides, and LDL-c and a significant increase of HDL-c of the rats treated animals with Omega-3 in groups 2 and 3 compared to lead acetate treated group (Table 4). Devasagayam et al. [40] suggested that oxidative modification of low-density lipoproteins (LDL-c) caused by reactive oxygen species results in the formation of foam cells which is the initial lesion of atherosclerosis. They also reported that LDL-c oxidation and atherogenesis can be inhibited by nutritional antioxidants. There are also epidemiological evidences and interventional studies to correlate higher level of antioxidant-rich food uptake with lower incidence of coronary heart disease [40].

The results of the present study demonstrated that lead acetate administration to female rats resulted in significant decrease of Hb, PCV, RBCs, and platelet count (PLT) of the rats treated with lead acetate in contrast to those in the negative control rats (Table 5). On the other hand, WBCs count of lead acetate treated rats was elevated compared to the negative control group, and these results are in agreement with those described by Kim et al. [41] and Simsek et al. [42]. However, Topashka-Ancheva et al. [43] showed that lead could damage the erythrocytes membrane resulting in hemolysis or decrease of blood iron level which may be the reason of decreasing the concentration of Hb and PCV. These hematological alterations might be also due to the effect of lead on the activity of *δ*-aminolevulinic acid dehydrogenase which acts as key enzyme of heme synthesis. Previous study reported that lead inhibits the conversion of coproporphyrinogen III to protoporphyrin IX leading to reduction in Hb production and shortening of life span of erythrocytes [44]. The results obtained in this study indicated that Omega-3 successfully maintained normal haematological parameters against the toxicity induced by lead acetate in female rats. Our data are in accordance with previous results which reported that supplementing rats with different doses of Omega-3 showed appreciable improvement in the haematological indices as evidenced by significant increase in Hb, PCV, and RBC counts and decrease in WBC counts [45].

Figures 1(a), 1(b), and 1(c) showed normal cellular architecture with distinct hepatic cells, sinusoidal spaces, and a central vein that were observed in the negative control group and Omega-3 at both doses-treated groups. Figure 1((d1) and (d2)) showed that lead acetate treatment induced severe histopathological alterations in liver; most of the intrahepatic blood vessels, especially the central veins, were dilated and congested. In addition, the hepatocytes lost their normal architecture and vacuolization with pyknotic nuclei appeared in the cytoplasm. These results are in agreement with the results of Abdel-Moneim et al. [46]. Our histological investigations of renal tissue revealed that Pb-acetate treatment results in progressive glomerular and tubular alterations. These findings are in agreement with the results of Abdel-Moneim et al. [34]. Omega-3 treatment caused a significant decrease in the histopathological changes induced by lead acetate in the liver and the kidney (Figures 1 and 2) and partially restored these changes in lead acetate plus Omega-3 treated groups.

RAPD and arbitrarily primed polymerase chain reaction technique (AP-PCR) are powerful tools for gene mapping, population, pedigree analysis, phylogenetic studies, and strain identification [47]. In addition, their use in surveying genomic DNA for evidence of various types of damage and mutation suggests that they may potentially form the basis of novel genotoxicological assays for the detection of DNA damage and mutations [48].

Previous studies have shown that changes in band patterns observed in DNA “fingerprint” analyses reflect DNA alterations from single base changes (point mutations) to complex chromosomal rearrangements [49, 50]. In this study, DNA damage induced by heavy metals was reflected by changes in RAPD profiles, disappearance of bands and appearance of new PCR products which occurred in the profiles generated by exposed rats to lead acetate. The present data showed that the RAPD-PCR method is useful for the screening and characterization of genomic regions that have undergone alterations as the result of lead acetate exposure. Several similar findings have been reported by Castaño and Becerril [51] and Liu et al. [52] that used RAPD-PCR to analyze the induced DNA damage. However, random amplified polymorphism of DNA (RAPD) showed distinct differences in animal groups exposed to lead acetate (group 4) and treated with Omega-3 (groups 2 and 3) at both doses (125 and 260 mg/kg body weight). Also, RAPD reflected the protective effect of Omega-3 on DNA. These results were consistent with those obtained by Elelaimy et al. [53] who reported that Omega-3 pre-/posttreatment to azathioprine showed high significance in reducing the percentage of DNA fragmentation compared to azathioprine treated mice.

## 5. Conclusion

In this study, the effect of lead acetate as one of the hazardous heavy metals was studied using biochemical tests; histopathological study and genomic analysis showed the high risk of lead toxicity through the exposure to lead acetate. Omega-3 acts as antioxidant compound and has protective and treatment effect versus lead toxicity, so it should be tested on other heavy metals and environmental toxic compounds. The biochemical analysis confirmed the free radical scavenging properties of Omega-3 as antioxidant compounds as well as the ability of Omega-3 to improve liver and kidney functions and haematological parameters. RAPD-PCR technique proved that it is a useful and effective technique to study the DNA damage due to lead toxicity through the mutation of DNA which can be studied through the absence or intensity of different pattern bands. So, the present results indicated that coadministration of Omega-3 had protective role against hepatotoxicity, renal toxicity, haematotoxicity, and genotoxicity induced by lead acetate.

## Figures and Tables

**Table 1:** PCR primers used in RAPD-PCR, GC%, and annealing temperature.

Primers	Sequence 5′ → 3′	GC%	Annealing temperature (°C/sec)
1	GTC CAT GCCA	60	30/60
2	ACA TCG CCCA	60	30/60
3	ATG CCC CTG T	60	30/60

**Table 2:** The activities of AST, ALT, ALP, LDH, levels of MDA, total protein, albumin, urea, and creatinine after lead exposure and Omega-3 treatment in serum of female rats.

Parameters	Experimental groups			
	(Group 1) −ve control	(Group 2) Omega-3 (125 mg/kg b.w.) + lead acetate	(Group 3) Omega-3 (260 mg/kg b.w.) + lead acetate	(Group 4) +ve control
AST (U/L)	56.69 ± 0.565^a^	58.63 ± 1.279^a^	51.35 ± 0.373^a^	104.27 ± 0.677^b^
ALT (U/L)	36.32 ± 0.276^a^	40.53 ± 0.260^a^	38.69 ± 0.416^a^	90.07 ± 0.612^b^
ALP (U/L)	42.43 ± 0.358^a^	48.74 ± 0.475^a^	41.44 ± 0.279^a^	87.77 ± 0.534^b^
LDH (U/L)	149.29 ± 0.703^a^	159.07 ± 0.845^a^	148.81 ± 0.845^a^	219.88 ± 1.082^b^
Total protein (gm/dL)	7.10 ± 0.115^a^	6.11 ± 0.244^a^	7.61 ± 0.217^a^	4.88 ± 0.103^b^
Albumin (g/dL)	3.96 ± 0.095^a^	3.75 ± 0.071^a^	4.05 ± 0.102^a^	2.66 ± 0.083^b^
Urea (mg/dL)	31.09 ± 0.225^a^	40.04 ± 0.715^a^	31.59 ± 0.450^a^	63.56 ± 0.962^b^
Creatinine (mg/dL)	0.38 ± 0.004^a^	0.47 ± 0.011^a^	0.38 ± 0.004^a^	0.96 ± 0.026^b^
MDA (nmol/mL)	17.08 ± 0.296^a^	18.81 ± 0.538^a^	17.47 ± 0.349^a^	51.99 ± 0.620^b^

Values are expressed as means ± SE; *n* = 7 for each treatment group. a, b indicate the significant results statistically; *P* < 0.05.

**Table 3:** The effect of Omega-3 and lead acetate on specific activity of liver and kidney antioxidant enzymes in female rats.

Parameters	Experimental groups			
	(Group 1) −ve control	(Group 2) Omega-3 (125 mg/kg b.w.) + lead acetate	(Group 3) Omega-3 (260 mg/kg b.w.) + lead acetate	(Group 4) +ve control
Liver				
GP_X_ (U/mg protein)	40.53 ± 0.240^a^	36.54 ± 0.909^a^	48.50 ± 0.341^a^	22.44 ± 0.487^b^
CAT (U/mg protein)	27.53 ± 0.554^a^	23.92 ± 0.583^a^	34.46 ± 0.743^a^	16.67 ± 0.489^b^
SOD (U/mg protein)	11.34 ± 0.355^a^	10.40 ± 0.346^a^	18.11 ± 0.495^b^	7.64 ± 0.303^c^
GSH (U/g tissue)	27.84 ± 0.312^a^	26.08 ± 0.446^a^	29.16 ± 0.271^a^	12.72 ± 0.303^b^
MDA (nmol/g tissue)	50.34 ± 0.259^a^	54.62 ± 0.288^a^	51.41 ± 0.282^a^	100.54 ± 0.322^b^
Kidney				
GP_X_ (U/mg protein)	30.64 ± 0.521^a^	27.43 ± 0.597^a^	39.89 ± 0.479^b^	21.54 ± 0.360^c^
CAT (U/mg protein)	53.17 ± 0.477^a^	46.97 ± 0.557^a^	63.30 ± 0.583^b^	20.41 ± 0.562^c^
SOD (U/mg protein)	98.92 ± 0.457^a^	81.99 ± 0.693^a^	98.60 ± 0.285^a^	41.75 ± 0.451^b^
GSH (U/g tissue)	53.81 ± 0.682^a^	50.39 ± 0.350^a^	55.01 ± 0.724^a^	28.51 ± 0.334^b^
MDA (nmol/g tissue)	22.19 ± 0.320^a^	37.60 ± 0.382^b^	22.94 ± 0.515^a^	51.82 ± 0.313^c^

Values are expressed as means ± SE; *n* = 7 for each treatment group. a, b, and c indicate the significant results statistically; *P* < 0.05.

**Table 4:** Serum lipid and lipoprotein profiles of female rats after lead exposure and Omega-3 treatment.

Parameters	Experimental groups			
	(Group 1) −ve control	(Group 2) Omega-3 (125 mg/kg b.w.) + lead acetate	(Group 3) Omega-3 (260 mg/kg b.w.) + lead acetate	(Group 4) +ve control
TL (mg/dL)	201.59 ± 2.215^a^	179.85 ± 1.879^a^	150.19 ± 0.745^b^	404.00 ± 9.398^c^
TG (mg/dL)	96.58 ± 0.706^a^	100.35 ± 0.671^a^	95.23 ± 0.686^a^	131.41 ± 0.541^b^
Cholesterol (mg/dL)	54.96 ± 1.665^a^	61.29 ± 0.854^a^	54.43 ± 1.269^a^	84.05 ± 2.194^b^
LDLC (mg/dL)	25.83 ± 0.438^a^	28.77 ± 0.431^a^	19.84 ± 0.399^a^	41.73 ± 0.470^b^
HDLC (mg/dL)	29.14 ± 0.688^a^	28.75 ± 0.573^a^	30.19 ± 0.531^a^	18.17 ± 0.472^b^

Values are expressed as means ± SE; *n* = 7 for each treatment group. a, b, and c indicate the significant results statistically; *P* < 0.05.

**Table 5:** Changes in hematological parameters of female rats treated after lead exposure and Omega-3 treatment.

Parameters	Experimental groups			
	(Group 1) −ve control	(Group 2) Omega-3 (125 mg/kg b.w.) + lead acetate	(Group 3) Omega-3 (260 mg/kg b.w.) + lead acetate	(Group 4) +ve control
Hb (g/dL)	14.74 ± 0.070^a^	13.70 ± 0.155^ a^	14.31 ± 0.107^a^	9.67 ± 0.153^b^
PCV (%)	41.14 ± 0.388^a^	40.25 ± 0.503^a^	42.84 ± 0.618^a^	31.86 ± 0.346^b^
RBCs (×10^12^ L^−1^)	5.67 ± 0.133^a^	4.97 ± 0.174^a^	5.96 ± 0.162^a^	3.74 ± 0.056^b^
WBCs (×10^9^ L^−1^)	3.97 ± 0.196^a^	4.98 ± 0.084^ab^	3.90 ± 0.076^a^	5.96 ± 0.130^b^
PLT (×10^12^ L^−1^)	255.14 ± 8.250^a^	217.57 ± 3.518^a^	268.29 ± 9.551^a^	116.57 ± 8.352^b^

Values are expressed as means ± SE; *n* = 7 for each treatment group. a, b indicate the significant results statistically, while ab may be significant or not significant; *P* < 0.05.

**Table 6:** Random primers showing polymorphism of DNA from liver of the four groups.

Primer code	Nucleotide sequence 5′ → 3′	Total number of amplified fragments	Number of monomorphic fragments	Number of polymorphic fragments	Fragments size range (bp)
1	GTC CAT GCCA	13	2	11	250–1300
2	ACA TCG CCCA	12	1	11	300–1500
3	ATG CCC CTG T	11	0	11	250–1700

Total		36	3	33	

**Table 7:** Agreement percentage of RAPD profile.

	gp1	gp2	gp3	gp4
gp1	100	81	75	64
gp2	81	100	78	72
gp3	75	78	100	89
gp4	64	72	89	100

## References

[1] Chatterjee MN, Rana S (2002). Metabolism of minerals and trace elements. *Textbook of Medical Biochemistry*.

[2] Markowitz M (2000). Lead poisoning. *Pediatrics in Review*.

[3] Courtois E, Marques M, Barrientos A, Casado S, López-Farré A (2003). Lead-induced downregulation of soluble guanylate cyclase in isolated rat aortic segments mediated by reactive oxygen species and cyclooxygenase-2. *Journal of the American Society of Nephrology*.

[4] Rahman S, Sultana S (2006). Chemopreventive activity of glycyrrhizin on lead acetate mediated hepatic oxidative stress and its hyperproliferative activity in Wistar rats. *Chemico-Biological Interactions*.

[5] Bolin CM, Basha R, Cox D (2006). Exposure to lead and the developmental origin of oxidative DNA damage in the aging brain. *FASEB Journal*.

[6] Farrag A-RH, Mahdy KA, Abdel Rahman GH, Osfor MM (2007). Protective effect of *Nigella sativa* seeds against lead-induced hepatorenal damage in male rats. *Pakistan Journal of Biological Sciences*.

[7] El-Nekeety AA, El-Kady AA, Soliman MS, Hassan NS, Abdel-Wahhab MA (2009). Protective effect of *Aquilegia vulgaris* (L.) against lead acetate-induced oxidative stress in rats. *Food and Chemical Toxicology*.

[8] Al-Saleh IAS (1994). The biochemical and clinical consequences of lead poisoning. *Medicinal Research Reviews*.

[9] Dressier J, Kim K-A, Chakraborti T, Goldstein G (1999). Molecular mechanisms of lead neurotoxicity. *Neurochemical Research*.

[10] Patrick L (2006). Lead toxicity part II: the role of free radical damage and the use of antioxidants in the pathology and treatment of lead toxicity. *Alternative Medicine Review*.

[11] Ademuyiwa O, Ugbaja RN, Rotimi SO (2007). Erythrocyte acetylcholinesterase activity as a surrogate indicator of lead-induced neurotoxicity in occupational lead exposure in Abeokuta, Nigeria. *Environmental Toxicology and Pharmacology*.

[12] Calviello G, Serini S (2010). *Dietary Omega-3 Polyunsaturated Fatty Acids and Cancer*.

[13] Shaikh IAA, Brown I, Wahle KWJ, Heys SD (2010). Enhancing cytotoxic therapies for breast and prostate cancers with polyunsaturated fatty acids. *Nutrition and Cancer*.

[14] Calder PC (2009). Polyunsaturated fatty acids and inflammatory processes: new twists in an old tale. *Biochimie*.

[15] Wall R, Ross RP, Fitzgerald GF, Stanton C (2010). Fatty acids from fish: the anti-inflammatory potential of long-chain omega-3 fatty acids. *Nutrition Reviews*.

[16] Edwards IJ, O’Flaherty JT (2008). Omega-3 fatty acids and PPAR *γ* in cancer. *PPAR Research*.

[17] Sun W-H, Chen G-S, Ou X-L (2009). Inhibition of COX-2 and activation of peroxisome proliferator-activated receptor *γ* synergistically inhibits proliferation and induces apoptosis of human pancreatic carcinoma cells. *Cancer Letters*.

[18] Wendel M, Heller AR (2009). Anticancer actions of omega-3 fatty acids—current state and future perspectives. *Anti-Cancer Agents in Medicinal Chemistry*.

[19] Koletzko B, Goulet O (2010). Fish oil containing intravenous lipid emulsions in parenteral nutrition-associated cholestatic liver disease. *Current Opinion in Clinical Nutrition and Metabolic Care*.

[20] Fassett RG, Gobe GC, Peake JM, Coombes JS (2010). Omega-3 polyunsaturated fatty acids in the treatment of kidney disease. *American Journal of Kidney Diseases*.

[21] Saravana Kumar A, Bhagya Deepthi K, Devi Vara Prasad M, Grace Mary P, Sujeeth Kumar S, Swathi M (2011). Evaluation of the protective effects of omega-3 fatty acids against methotrexate induced testicular toxicity in male albino mice. *International Journal of Phytopharmacology*.

[22] Ponce-Canchihuamán JC, Pérez-Méndez O, Hernández-Muñoz R, Torres-Durán PV, Juárez-Oropeza MA (2010). Protective effects of *Spirulina maxima* on hyperlipidemia and oxidative-stress induced by lead acetate in the liver and kidney. *Lipids in Health and Disease*.

[23] Bancroft D, Gamble M (2002). *The Theory and Practice of Histological Techniques*.

[24] Bardakci F, Skibinski DOF (1994). Application of the RAPD technique in tilapia fish: species and subspecies identification. *Heredity*.

[25] Williams JGK, Hanafey MK, Rafalski JA, Tingey SV (1993). Genetic analysis using random amplified polymorphic DNA markers. *Methods in Enzymology*.

[26] Steel GD, Torrie JH (1981). *Principles and Procedures of Statistics*.

[27] Herman DS, Geraldine M, Venkatesh T (2009). Influence of minerals on lead-induced alterations in liver function in rats exposed to long-term lead exposure. *Journal of Hazardous Materials*.

[28] Ibrahim NM, Eweis EA, El-Beltagi HS, Abdel-Mobdy YE (2012). Effect of lead acetate toxicity on experimental male albino rat. *Asian Pacific Journal of Tropical Biomedicine*.

[29] Mehana EE, Meki ARMA, Fazili KM (2012). Ameliorated effects of green tea extract on lead induced liver toxicity in rats. *Experimental and Toxicologic Pathology*.

[30] Yousef MI (2004). Aluminium-induced changes in hemato-biochemical parameters, lipid peroxidation and enzyme activities of male rabbits: protective role of ascorbic acid. *Toxicology*.

[31] Gaskill CL, Miller LM, Mattoon JS (2005). Liver histopathology and liver and serum alanine aminotransferase and alkaline phosphatase activities in epileptic dogs receiving phenobarbital. *Veterinary Pathology*.

[32] Abdou HM, Newairy AA (2006). Hepatic and reproductive toxicity of lead in female rats and attenuation by flaxseed lignans. *Journal of Medical Research Institute*.

[33] Garba SH, Adelaiye AB, Mshelia LY (2007). Histopathological and biochemical changes in the rats kidney following exposure to a pyrethroid based mosquito coil. *Journal of Applied Sciences Research*.

[34] Abdel-Moneim AE, Dkhil MA, Al-Quraishy S (2011). The potential role of flaxseed oil on lead acetateinduced kidney injure in adult male Albino rats. *African Journal of Biotechnology*.

[35] Attia AM, El-Banna SG, Nomeir FR, El-Basser MIA (2011). Lindane-induced biochemical perturbations in rat serum and attenuation by omega-3 and *Nigella sativa* seed oil. *Indian Journal of Biochemistry and Biophysics*.

[36] Pauwels EKJ, Kostkiewicz M (2008). Fatty acid facts, part III: cardiovascular disease, or, a fish diet is not fishy. *Drug News and Perspectives*.

[37] Asaad HR, Aziz FM (2012). Protective role of omega-3 fish oil against the toxicity of ifosfamide in male rats. *Jordan Journal of Biological Sciences*.

[38] Attia AM, Nasr HM (2009). Dimethoate-induced changes in biochemical parameters of experimental rat serum and its neutralization by black seed (*Nigella sativa* L.) oil. *Slovak Journal Animal Science*.

[39] Newairy A-SA, Abdou HM (2009). Protective role of flax lignans against lead acetate induced oxidative damage and hyperlipidemia in rats. *Food and Chemical Toxicology*.

[40] Devasagayam TPA, Tilak JC, Boloor KK, Sane KS, Ghaskadbi SS, Lele RD (2004). Free radicals and antioxidants in human health: current status and future prospects. *Journal of Association of Physicians of India*.

[41] Kim R, Rotnitzky A, Sparrow D, Weiss ST, Wager C, Hu H (1996). A longitudinal study of low-level lead exposure and impairment of renal function: the normative aging study. *Journal of the American Medical Association*.

[42] Simsek N, Karadeniz A, Kalkan Y, Keles ON, Unal B (2009). Spirulina platensis feeding inhibited the anemia- and leucopenia-induced lead and cadmium in rats. *Journal of Hazardous Materials*.

[43] Topashka-Ancheva M, Metcheva R, Teodorova S (2003). Bioaccumulation and damaging action of polymetal industrial dust on laboratory mice Mus musculus alba II. Genetic, cell, and metabolic disturbances. *Environmental Research*.

[44] Klassen CD (2001). *Casarett and Doull's Toxicology: The Basic Science of Poisons*.

[45] Ndem JI, Akpanabiatu MI, Essien EU (2008). Effects of seafoods (Periwinkle, Bonkafish and Crayfish) and vegetable oils enriched meal on cardiovascular disease. *Pakistan Journal of Nutrition*.

[46] Abdel-Moneim AE, Dkhil MA, Al-Quraishy S (2011). The redox status in rats treated with flaxseed oil and lead-induced hepatotoxicity. *Biological Trace Element Research*.

[47] Grayson TH, Atienzar FA, Alexander SM, Cooper LF, Gilpin ML (2000). Molecular diversity of *Renibacterium salmoninarum* isolates determined by randomly amplified polymorphic DNA analysis. *Applied and Environmental Microbiology*.

[48] Shimada A, Shima A (1998). Combination of genomic DNA fingerprinting into the medaka specific- locus test system for studying environmental germ-line mutagenesis. *Mutation Research*.

[49] White JJ, Neuwirth H, Dennis Miller C, Schneider EL (1990). DNA alterations in prostatic adenocarcinoma and benign prostatic hyperplasia: detection by DNA fingerprint analyses. *Mutation Research*.

[50] Atienzar FA, Conradi M, Evenden AJ, Jha AN, Depledge MH (1990). Qualitative assessment of genotoxicity using random amplified polymorphic DNA: comparison of genomic template stability with key fitness parameters in *Daphnia manna* exposed to benzo (a) pyrene. *Environmental Toxicology and Chemistry*.

[51] Castaño A, Becerril C (2004). In vitro assessment of DNA damage after short- and long-term exposure to benzo(a)pyrene using RAPD and the RTG-2 fish cell line. *Mutation Research—Fundamental and Molecular Mechanisms of Mutagenesis*.

[52] Liu W, Li PJ, Qi XM (2005). DNA changes in barley (*Hordeum vulgare*) seedlings induced by cadmium pollution using RAPD analysis. *Chemosphere*.

[53] Elelaimy IA, Elfiky SA, Hassan AM, Ibrahim HM, Elsayad RI (2012). Genotoxicity of anticancer drug Azathioprine (Imuran): role of omega-3 (*ω*-3) oil as protective agent. *Journal of Applied Pharmaceutical Science*.

